# The utility of ^18^F-FDG PET and PET/CT in the diagnosis and staging of chondrosarcoma: a meta-analysis

**DOI:** 10.1186/s13018-020-01748-w

**Published:** 2020-06-22

**Authors:** Qingyu Zhang, Yongming Xi, Dong Li, Zenong Yuan, Jun Dong

**Affiliations:** 1 Department of Orthopedics, Shandong Provincial Hospital Affiliated to Shandong First Medical University, No.324, Road Jing Wu Wei Qi, Jinan, 250021 Shandong China; 2 Department of Spine Surgery, Affiliated Hospital of Qingdao University, 16 Jiangsu Road, Qingdao, 266000 Shandong Province China

**Keywords:** Chondrosarcoma, ^18^F-FDG PET, PET/CT, Diagnosis, Meta-analysis

## Abstract

**Objective:**

Chondrosarcoma is the second most common primary bone sarcoma; however, unlike other tumors, the biopsy cannot easily make a definite diagnosis or predict the histological grade. This meta-analysis was performed to evaluate the utility of ^18^F-FDG PET and PET/CT to differentiate chondrosarcoma from benign cartilaginous lesions and to predict the histopathological grade of chondrosarcoma.

**Material and methods:**

A comprehensive search was performed in three electronic databases including Medline/PubMed, the Cochrane Library and Embase to retrieve diagnostic studies evaluating the role of ^18^F-FDG PET or PET/CT for appraising the status of chondrosarcoma. Reference lists of related articles were also scrutinized manually. Useful data were extracted to calculate the pooled sensitivity, specificity, positive likelihood ratio (PLR), negative likelihood ratio (NLR), diagnostic odds ratio (DOR), the summary receiver operating characteristic curve (sROC), and the area under the curve (AUC) of ^18^F-FDG PET or PET/CT in diagnosing chondrosarcoma, and pooled weighted mean differences (WMD) of maximum standardized uptake value (SUVmax) between different entities of cartilaginous neoplasms by using Stata 19.0.

**Results:**

A total of twelve studies provided sufficient data for the quantitative analysis. For the diagnosis of chondrosarcoma, the pooled sensitivity, specificity, and DOR of ^18^F-FDG PET were 0.84 (95% confidence interval [CI] 0.46 to 0.97), 0.82 (95% CI 0.55 to 0.94), and 24.244 (95% CI 1.985 to 96.148), respectively while those of ^18^F-FDG PET/CT were 0.94 (95% CI 0.86 to 0.97), 0.89 (95% CI 0.82 to 0.93), and 112.999 (95% CI 41.341 to 308.866), respectively. The pooled WMD of SUVmax were − 0.89 (95% CI −1.67 to −0.10) between benign cartilaginous lesions and grade 1 (G1) chondrosarcoma, −1.94 (95% CI −2.76 to −1.12) between G1 and grade 2 (G2) chondrosarcoma, and − 2.37 (95% CI −5.79 to 1.05) between G2 and grade 3 (G3) chondrosarcoma.

**Conclusions:**

In a word, ^18^F-FDG PET/CT revealed excellent accuracy in the diagnosis of chondrosarcoma and might assist in clinical decision-making. Meanwhile, although SUVmax alone showed restricted ability to differentiate benign cartilaginous lesions and G1 chondrosarcoma, as well as between G2 and G3 chondrosarcoma, it can identify intermediate/high-grade chondrosarcoma from low-grade ones.

**Level of evidence:**

Level I evidence, a summary of meta-analysis

## Introduction

Chondrosarcoma is the second most common primary malignant bone sarcoma characterized by the production of atypical cartilage matrix and invasive growth inside the pre-existing cortical and medullary bone tissue [1]. This malignant disorder could be further subcategorized to low-grade (G1), intermediate-grade (G2), high-grade (G3), and dedifferentiated chondrosarcoma, which manifest diverse histological features and clinical behaviors [2]. G1 chondrosarcoma has little risk of metastasis and excellent prognosis [3] while an unfavorable outcome is generally associated with G2 and G3 chondrosarcoma, revealing 5-year cumulative survival rates being 63-92% and 39-77%, respectively [3]. Dedifferentiated chondrosarcoma exhibits a biphasic differentiated nature (the conventional chondrosarcoma and the high-grade, non-cartilage-producing sarcoma) of tumor cells, predisposing patients with the worst prognosis [4]. The widely-accepted regimen for managing benign cartilaginous neoplasms (e.g., enchondroma and exostosis) is the follow-up, and marginal excision when symptoms arise [5]. Alternative options advocated for G1 chondrosarcoma include rigorous follow-up until the lesion progresses, wide excision or curettage, albeit the last approach may be accompanied by relapse [6]. Besides, neoadjuvant chemotherapy before surgery can be reserved for dedifferentiated chondrosarcoma [7] and radiotherapy (as a palliative treatment) for unresectable lesions [7, 8].

Therefore, the optimal therapeutic strategy of cartilaginous bone neoplasms should be established on the accurate diagnosis and staging. Although chondrosarcoma normally presents with increased pain, these symptoms and signs can be nonspecific and lead to a misdiagnosis of other musculoskeletal disorders such as osteomyelitis and osteoarthritis [2]. Up to date, the classification of this heterogeneous entity mainly relies on comprehensive considering clinical, imaging, and histological information, but a definitive diagnosis and grading are often difficult to achieve due to the discrepant interpretation among observers [9]. Meanwhile, tissue samples acquired by core needle biopsy may not be representative of the entire cartilaginous lesion, leading to underestimating the degree of dedifferentiation [10]. According to Laitinen et al.’s report enrolling 343 patients with osteochondroma, the concordance between the preoperative biopsy grading and the final post-surgical histologic diagnosis was observed in only 43% cases [10]. Conventional imaging methods such as X-ray, computed tomography (CT) scan, magnetic resonance imaging (MRI), and bone scintigraphy have been used as adjuvants for evaluating patients with suspected chondrosarcoma but often result in false negatives or false positives [11, 12].

^18^F-fluorodeoxyglucose (^18^F-FDG) avidity provides useful information regarding tumor biology and sarcomatous transformation by depicting glucose metabolism and identifying hypermetabolic foci [13]. Meanwhile, a hybrid of ^18^F-FDG positron emission tomography (PET) and computed tomography (CT) combines metabolic and anatomic data and may continue to improve diagnostic efficiency [13]. A series of studies have investigated the utility of ^18^F-FDG PET or PET/CT in the diagnosis and staging of chondrosarcoma and revealed contradictory conclusions [14–16]. A systematic review [17] published in 2017 attempted to summarize the optimal maximum standardized uptake value (SUVmax) to differentiate different groups of cartilaginous bone sarcoma but the reliability of its result was compromised due to several flaws. First, only 8 studies involving 166 chondroid neoplasms were listed. Second, the investigators did not appraise the diagnostic accuracy of ^18^F-FDG PET or ^18^F-FDG PET/CT for chondrosarcoma. Third, the variations of ^18^F-FDG avidity between different chondroid neoplasms were not compared with a standard approach of evidence-based medicine. Multiple high-quality studies [14–16] on this topic were available in recent years and the current investigation aimed to further assess the ability of ^18^F-FDG PET and PET/CT to diagnose chondrosarcoma and to predict the histological grade by performing a meta-analysis.

## Materials and methods

The methodological approach described later complied with the Preferred Reporting Items for a Systematic Review and Meta-analysis of Diagnostic Test Accuracy Studies (PRISMA-DTA) [18]. Ethical approval or informed consent was waived given that all data were retrieved from published literature. Database searching, eligibility assessment, data extraction, and methodological quality evaluation were performed by two investigators (QY Zhang and J Dong) independently and repeatedly. Any disagreement was resolved through discussion and consensus among the research team.

### Search strategy

A systematic literature search was performed in three electronic databases including Medline/PubMed, Embase, and Cochrane Library using combinations of following keywords: (“PET” OR “positron emission tomography”) AND (“chondroid” OR “cartilaginous” OR “cartilage” OR “chondrosarcoma”) without language or publication period limitations. Meanwhile, reference lists of relevant articles (diagnostic studies, reviews, meta-analyses, and editorials) were carefully checked to avoid missing additional eligible studies.

### Inclusion and exclusion criteria

Studies eligible for our meta-analysis must confirm the following criteria: (1) studies assessing the diagnostic or staging value of ^18^F-FDG PET or PET/CT in cartilaginous neoplasms (benign cartilaginous tumors and/or chondrosarcoma); (2) final diagnosis was confirmed by histopathological examination for chondrosarcoma, and follow-up or histopathological examination for benign lesions (3) raw data such as the number of true-positive (TP), false positive (FP), false negative (FN), and true negative (TN) cases, or maximum standardized uptake value (SUVmax) of enrolled participants were provided. Exclusion criteria for this meta-analysis included (1) animal studies; (2) studies with less than five participants, and (3) posters displayed at the congress, abstracts, letters, and comments due to the lack of essential information.

If more than one article contained overlapping data, the most comprehensive or recent one was included.

### Data extraction and methodological quality evaluation

Following data were extracted from original articles and entered into a standardized excel file: first author’s surname, publication year, study design, number and characteristics of participants (i.e., age and gender), tumor histology, reference methods, details of index tests (i.e., ^18^F-FDG PET or PET/CT, injection dose and methods of analysis), SUVmax, and final diagnosis. Numbers of TP, FP, TN, and FN were extracted directly or recalculated through data obtained from original articles. The risk of bias of included studies was appraised by using the QUADAS-2 tool [19], which consisted of four key domains (i.e., patient selection, index test, reference standard, and flow and timing) involving 14 questions. These questions were answered with “yes” for a low risk of bias, “no” for a high risk of bias, and “unclear” if associated information was not clearly depicted [19].

### Statistical analysis

Pooled sensitivity, specificity, positive likelihood ratio (PLR), negative likelihood ratio (NLR), and diagnostic odds ratio (DOR) were calculated using the bivariate meta-analysis framework (a random-effects model). In addition, summarized receiver operating characteristic (sROC) curves were constructed, with a larger area under the curve (AUC) indicating a better diagnostic accuracy of tests. Meanwhile, pooled weighted mean difference (WMD) as well as related 95% confidence intervals (CIs) was generated to evaluate continuous data (SUVmax), and a 95% CIs not covering 0 revealed a difference with statistical significance. Heterogeneity among included studies was assessed using the *I*^*2*^ statistics. An *I*^*2*^ value of 0% implied no observed heterogeneity, and > 50% suggested substantial heterogeneity. All data were analyzed using Stata version 19.0 (StataCorp, College Station, TX).

## Results

### Study selection and description

**Fig. 1:**
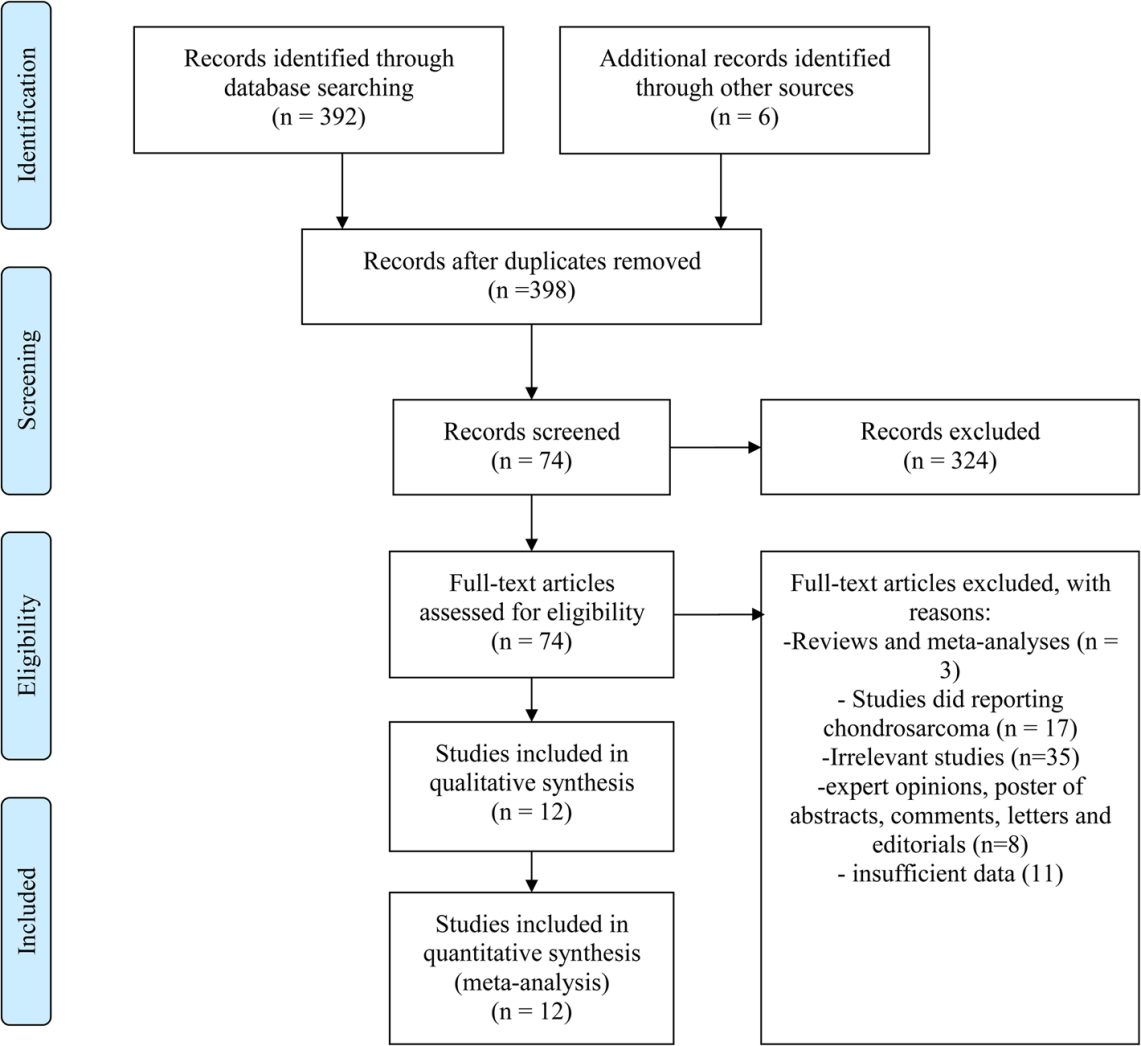
Selection process of included studies. Three hundred ninety-eight records were retrieved by database searching and screening of reference lists. After assessment of titles and abstracts, and then full-reading of remaining records, a total of 12 studies were included in the quantitative analysis

By searching electronic databases and reviewing reference lists of relevant publications, a total of 398 records were retrieved, among which 324 apparently ineligible articles were firstly discarded by screening titles and abstracts. Subsequently, full texts of the remaining ones were downloaded and scrutinized against the predefined criteria. Eventually, twelve [12, 14–16, 20–27] studies were included in the quantitative analysis. The selection process and reasons for exclusion were described in Fig. 1.

### Study characteristics

**Fig. 2:**
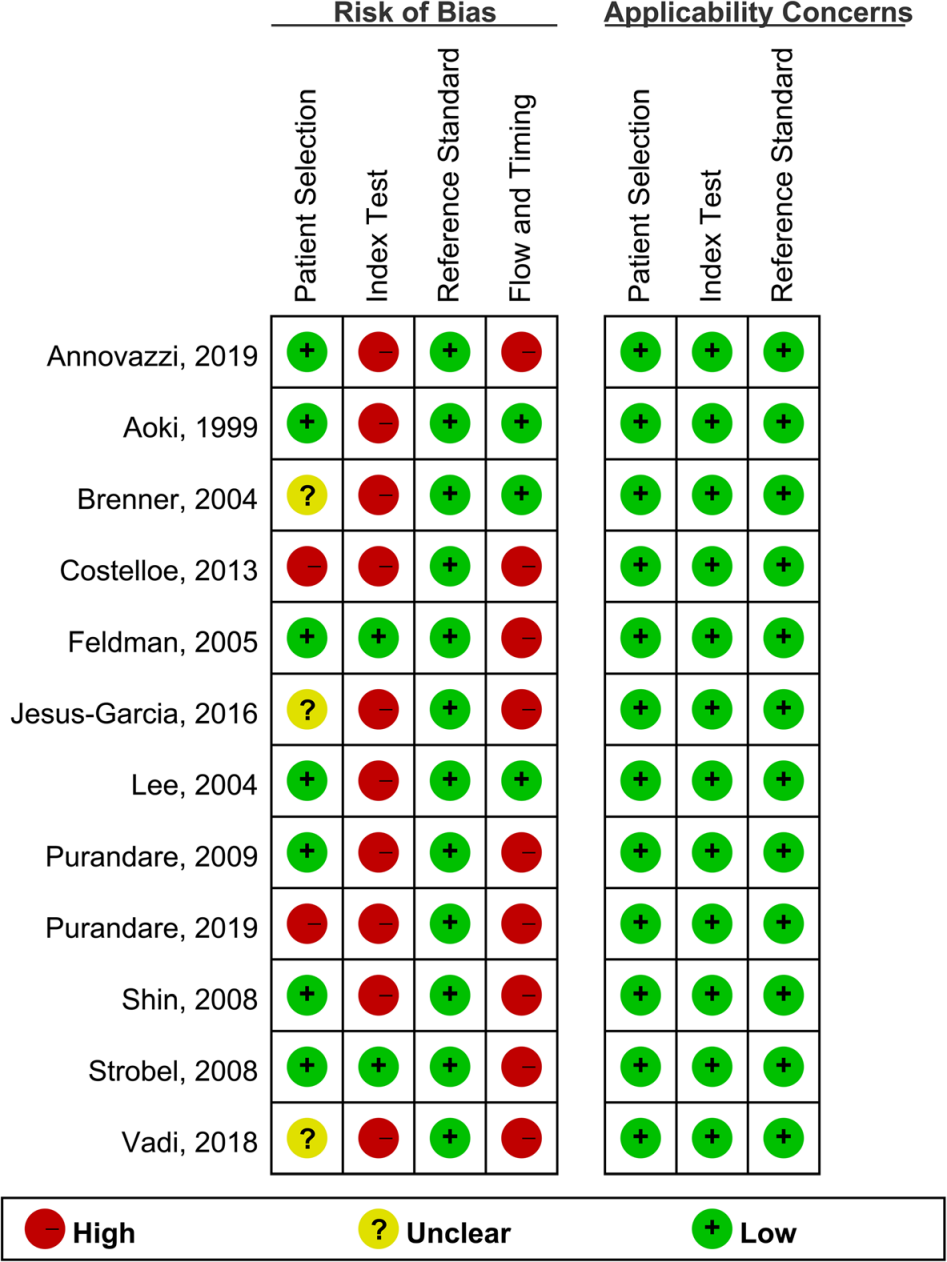
Quality assessment of included studies using QUADAS-2 tool criteria. Red in figure indicates high risk, yellow represents unclear risk, and green means low risk

All included studies are published in English and among them, three [12, 23, 27] were prospective, whereas nine [14–16, 20–22, 24–26] were retrospective. Sizes of these studies ranged from 7 to 95 and a total of 375 participants with suspected chondrosarcoma were involved. Only one [14] study investigated the diagnostic value of both ^18^F-FDG PET and PET/CT for chondrosarcoma. Six [12, 14, 16, 24–26] studies provided sufficient data about the distribution of SUVmax in different stages of chondrosarcoma. Main characteristics of the included studies were summarized in Table 1. As for the risk of bias, only two [23, 24] studies were judged as low risk in the section of index tests because the rest did not predefine the cutoff value of SUVmax for diagnosing chondrosarcoma. Meanwhile, nine [12, 14–16, 20–24] studies were judged as high risk of bias in flow and timing for the lack of a uniform reference test for all enrolled participants, which was hard to achieve and maybe ethically questionable in some situations. In nine studies [12, 14–16, 20–24], the golden standard to diagnose cartilaginous tumors was biopsy for those highly suggestive of malignancy and follow-up for those with benign manifestation. Results of the risk of bias assessment were summarized in Fig. 2.

**Table 1:** Basic characteristics of included studies

Author	Year	Country	Design	Number of subjects	No. of patients with chondrosarcoma	Gender (M/F)	Age (mean, years)	Type of cartilaginous bone neoplasms	Inclusion interval	Imaging	Cutoff value of SUV_max_	Injected dose	Reference test
Annovazzi et al. [14]	2019	Italy	R	95	60	40/55	53 ± 15	Enchondroma and chondrosarcoma	2011-2017	PET and PET/CT	2.6	5 MBq/kg	Post-surgical histology diagnosis, clinical and radiologic follow-up
Purandare et al. [15]	2019	India	R	66	40	NR	NR	Chondrosarcoma, enchondroma, and osteochondroma	2008.1-2018.6	PET/CT	3.1	5 MBq/kg	Post-surgical or a bioptic-histological diagnosis, clinical and radiologic follow-up
Vadi, et al [16]	2018	India	R	31	NR	20/21	42.19 (17-69)	Chondrosarcoma	2010.1-2016.12	PET/CT	NR	300-370 MBq	Pooled information from histological results and clinical or imaging follow-up
Jesus-Garcia et al. [12]	2016	Brazil	P	36	19	12/24	44 (21-68)	Enchondroma and chondrosarcoma	2009.10-2015.5	PET/CT	2.2	3.7 MBq/kg	Post-surgical histological diagnosis, clinical and radiologic follow-up
Costelloe et al. [20]	2013	USA	R	18	5	NR	NR	Enchondroma and chondrosarcoma	2007.1.1-2010.10.1	PET/CT	NR	370 MBq or 555-740 MBq	Pooled information from histological results and clinical or imaging follow-up
Purandare et al. [21]	2009	India	R	12	4	7/5	29.9 ± 11.6 (15-50)	Osseocartilaginous tumor, chondrosarcoma	2005-2007	PET/CT	1.3/3.3	370 MBq	Pooled information from histological results and clinical or imaging follow-up
Shin et al. [22]	2008	South Korea	R	12	6	NR	NR	Chondroblastoma, chondroma, enchondroma, and chondrosarcoma	2004.5-2007.6	PET/CT	3.8	8.14 MBq/kg	Pooled information from histological results and clinical or imaging follow-up
Strobel et al. [23]	2008	Switzerland	P	7	3	NR	NR	Chondrosarcoma, osteochondroma, chondroblastoma, and enchondroma	NR	PET	2.5	350-400 MBq	Pooled information from histological results and clinical or imaging follow-up
Feldman et al. [24]	2005	USA	R	29	18	9/20	53 ± 17.58 (11-85)	Enchondroma, osteochondroma, and chondrosarcoma	2000-2003	PET	2	5.18 MBq/kg	Pooled information from histological results and clinical or imaging follow-up
Brenner et al. [25]	2004	USA	R	31	31	17/14	50.4 ± 14.3 (23-85)	Chondrosarcoma	1995.5-2002.5	PET	NR	370 MBq	Post-surgical histology diagnosis
Lee et al. [26]	2004	USA	R	27	13	18/9	NR	Enchondroma, osteochondroma, and chondrosarcoma	1999-2002	PET	NR	5.18 MBq/kg	Histopathologic examination
Aoki et al. [27]	1999	Japan	P	11	5	4/7	44.3 ± 21.0 (12-78)	Enchondroma, osteochondroma, and chondrosarcoma	1997.8-1998.12	PET	1.3	5 MBq/kg	Histopathologic examination

*P* prospective; *R* retrospective; *NR* not reported; *PET* positron emission tomography; *CT* computed tomography

### Accuracy of ^18^F-FDG PET for the diagnosis of chondrosarcoma

**Fig. 3:**
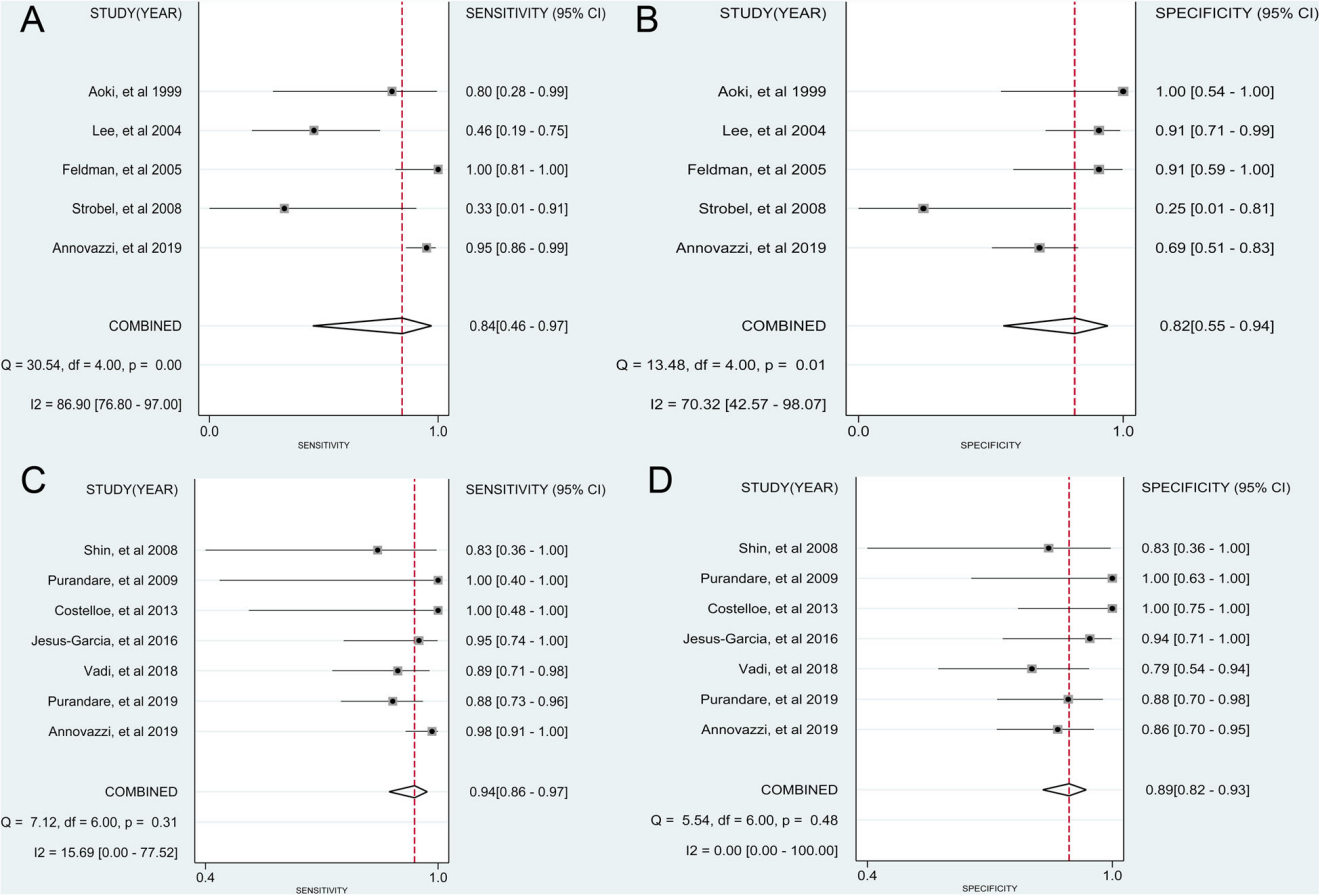
Forest plots of the pooled sensitivity and specificity with corresponding 95% confidence interval for the diagnosis of chondrosarcoma. **a** Pooled sensitivity of ^18^F-FDG PET. **b** Pooled specificity of ^18^F-FDG PET. **c** Pooled sensitivity of ^18^F-FDG PET/CT. **d** Pooled specificity of ^18^F-FDG PET/CT. CI, confidence interval

**Fig. 4:**
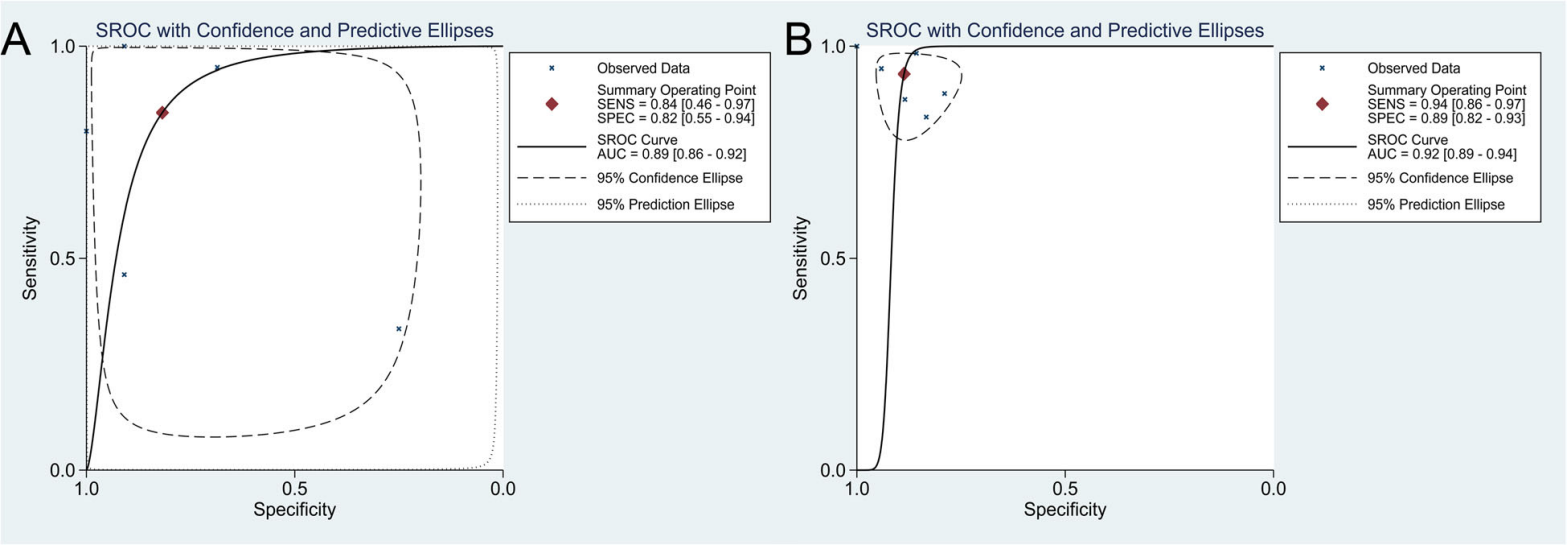
The pooled DOR with corresponding 95% confidence interval for the diagnosis of chondrosarcoma. **a** Pooled DOR of ^18^F-FDG PET. **b** Pooled DOR of ^18^F-FDG PET/CT. CI, confidence interval; DOR, diagnostic odds ratio

Six [14, 23, 24, 26, 27] studies provided data about the diagnostic accuracy of ^18^F-FDG PET for chondrosarcoma. As shown in Fig. 3a and b, the pooled sensitivity and specificity of ^18^F-FDG PET for diagnosing chondrosarcoma were 0.84 (95% CI, 0.46 to 0.97) and 0.82 (95% CI, 0.55 to 0.94), respectively. The pooled PLR, NLR, and DOR were 4.633 (95% CI, 1.443 to 14.875), 0.191 (95% CI, 0.037 to 0.986) and 24.244 (95% CI, 1.985 to 296.148), respectively, while the AUC was 0.89 (95% CI, 0.86 to 0.92) (Fig. 4a). The *I*^2^ statistics for sensitivity and specificity values were 86.90% (95% CI, 76.80 to 97.00%) and 70.32% (95% CI, 42.57 to 98.07%), respectively, which indicated that substantial heterogeneity existed among included studies.

### Accuracy of ^18^F-FDG PET/CT for the diagnosis of chondrosarcoma

Seven [12, 14–16, 20–22] studies provided data about the diagnostic accuracy of ^18^F-FDG PET/CT for chondrosarcoma. As shown in Fig. 3c and d, the pooled sensitivity and specificity of ^18^F-FDG PET/CT for diagnosing chondrosarcoma were 0.94 (95%CI, 0.86 to 0.97) and 0.89 (95% CI, 0.82 to 0.93), respectively. The pooled PLR, NLR, and DOR were 8.265 (95% CI, 5.012 to 13.628), 0.073 (95% CI, 0.034 to 0.157), and 112.999 (95% CI, 41.341 to 308.866), respectively, while the AUC was 0.92 (95% CI, 0.89 to 0.94) (Fig. 4b). The *I*^*2*^ statistics for sensitivity and specificity values were 15.79% (95% CI, 0 to 77.52%) and 0% (95% CI, 0 to 100%), respectively, indicating that no substantial heterogeneity existed among included studies.

### Accuracy of ^18^F-FDG avidity for the staging of chondrosarcoma

**Fig. 5:**
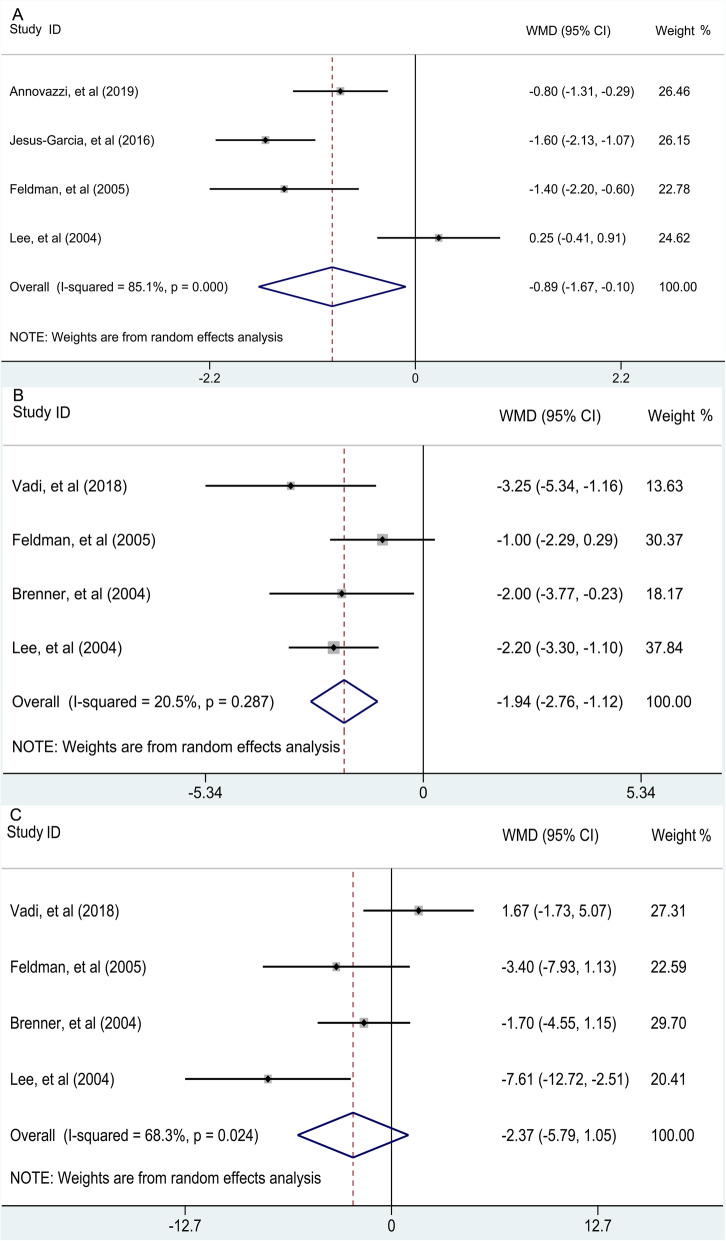
Forest plot of the comparison about SUVmax between (**a**) benign cartilaginous lesion and G1 chondrosarcoma, (**b**) G1 and G2 chondrosarcoma, and (**c**) G2 and G3 chondrosarcoma. WMD, weighted mean difference; CI, confidence interval

Four [12, 14, 24, 26] studies recorded the ability of ^18^F-FDG avidity to make a distinction between benign cartilaginous lesions and G1 chondrosarcoma. The combined results (pooled WMD = −0.89 95% CI, −1.67 to −0.10, *p* = 0.027; *I*^2^ statistic = 85.1%, *p* for heterogeneity < 0.001) suggested that SUVmax of benign cartilaginous lesions were slightly lower than that of G1 chondrosarcoma (Fig. 5a). Four [16, 24–26] studies recorded the ability of ^18^F-FDG avidity to differentiate between G1 and G2 chondrosarcoma. The SUVmax of G1 chondrosarcoma was significantly lower than that of G2 chondrosarcoma (pooled WMD = −1.94 95% CI, −2.76 to −1.12, *p* < 0.001; *I*^2^ statistic = 20.5%, *p* for heterogeneity = 0.287) (Fig. 5b). Meanwhile, four [16, 24–26] studies recorded the ability of ^18^F-FDG avidity to differentiate between G2 and G3 chondrosarcoma. The pooled results revealed that there was no significant difference of SUVmax between G2 and G3 chondrosarcoma (pooled WMD = −2.37 95% CI, −5.79 to 1.05, *p* = 0.174; *I*^2^ statistic = 68.3%, *p* for heterogeneity = 0.024) (Fig. 5c).

## Discussion

An optimal therapeutic strategy (observation or active treatment) for chondrosarcoma relies on not only the prompt identification but also the correct differentiation between high/intermediate-grade and low-grade ones. Besides fine needle aspiration, imaging examination should be carried out to decide whether invasive procedures are needed and whether the histology and imaging findings are concordant. In this study, by comprehensively reviewing eligible studies and adapting multiple statistic methods, it is demonstrated that overall ^18^F-FDG PET/CT has a higher accuracy to differentiate chondrosarcoma from benign cartilaginous lesions (pooled DOR = 112.999, 95% CI, 41.341 to 308.866) in comparison with^18^F-FDG PET (pooled DOR = 24.244; 95% CI, 1.985 to 296.148). To our knowledge, this is the first meta-analysis presenting the relatively accurate diagnostic efficacy of ^18^F-FDG PET (sensitivity = 0.84; specificity = 0.82) and PET/CT (sensitivity = 0.94; specificity = 0.89) for the diagnosis of chondrosarcoma.

Generally, it was reported that benign chondroid lesions such as enchondromas and osteochondromas are not FDG avid [14–16]. According to the pooled results, although there was a statistically significant WMD of SUVmax between G1 chondrosarcoma and benign cartilaginous lesions (pooled WMD = −0.89, 95% CI, −1.67 to −0.10), differential diagnosis of these two variants can be challenging owing to their histologically analogical nature and partially overlapped SUVmax. The confidence intervals of the pooled sensitivity (95% CI, 0.46-0.97) and specificity (95% CI, 0.55-0.94) were relatively wide, which was possibly on account of the small number of included studies, the little scale of sample size, and the significant between-study heterogeneity. Metabolic data alone is not substantial enough to provide robust data for the evaluation of chondrosarcoma. Meanwhile, only two [23, 24] studies pre-defined the cutoff value of SUVmax and it may not be possible to arrive a universally acknowledged SUVmax threshold (ranging from 1.3 to 3.1 in retrieved articles) that explicitly distinguish benign from malignant cartilaginous tumors [14, 23, 24, 26, 27]. A chondroid neoplasm with a SUVmax value of 1.5 would be considered as a benign tumor according to Jesus-Garcia et al.’ criteria [12] but malignancy in Aoki et al.’s study [27]. Institution-specific threshold established through the cooperation of nuclear medicine specialists, orthopedic surgeons, and pathologists was recommended in the clinical practice.

The combination of CT with ^18^F-FDG PET (^18^F-FDG PET/CT) facilitates the analysis of the volume and aggressive characteristics of the cartilaginous tumor and therefore may increase the diagnostic efficacy [14]. The size of cartilaginous tumors was closely correlated with their histologic grade; the majority of intermediate/high-grade chondrosarcoma (79.3%) was 5 cm or more in the maximum diameter [28]. Meanwhile, if there were signs of invasion (i.e., cortical bone invasion, periosteal reaction, bone expansion, periostitis, and extraosseous soft tissue), it is very likely that these cartilaginous lesions are chondrosarcomas [2]. In addition, CT scans of other parts of the body, especially the chest, are crucial for conducting a comprehensive staging of patients with chondrosarcoma [1, 2]. Promising pooled sensitivity (94%) and specificity (89%) of ^18^F-FDG PET/CT for diagnosing chondrosarcoma were revealed in the current study; meanwhile, the relatively narrower 95% confidence intervals (0.86-0.97 and 0.82-0.93, respectively) and smaller heterogeneity (*I*^2^ = 15.69% and 0%, respectively) in comparison with those of ^18^F-FDG PET indicated that these results were stable and persuasive. Whenever possible, ^18^F-FDG PET/PET, instead of ^18^F-FDG PET should be performed in patients with a suspicion of chondrosarcoma. However, PET/CT is not without disadvantages. Besides the additional radiation exposure associated with computed tomography, PET/CT is a costly and labor-consuming procedure that is not yet available in all hospitals [12]. Moreover, unlike thin-section dedicated CT, the CT section integrated with PET behaves poorly in presenting fine details such as depth of scalloping [14]. For the aim of getting a definitive diagnosis of a cartilaginous neoplasm, all available information such as patient age, clinical manifestation, lesion location, and the tumor growth rate must be taken into consideration [1, 2]. Creating a score that comprehensively summarizes these data as well as imaging and nuclear medicine findings will shed new light on the noninvasive diagnosis of cartilaginous neoplasms.

The current study was also designed to investigate the potential role of SUVmax to evaluate chondrosarcoma grading. The biological behavior of different chondrosarcoma entities varies greatly and the 2013 WHO classification of bone and soft tissue sarcoma clearly separates locally aggressive chondrosarcoma (atypical cartilaginous tumor or G1 chondrosarcoma) from definitely malignant cartilaginous tumors (G2 and G3 chondrosarcoma) [2]. It was postulated that the SUVmax values increased with the tumor grade [24] and the pooled result indeed demonstrated that ^18^F-FDG PET could accurately discriminate low-grade chondrosarcoma from intermediate-grade one with a pooled WMD of SUVmax being −3.25, albeit there was an evident overlap in SUVmax values between G2 and G3 chondrosarcoma. Significant heterogeneity of included studies (*I*^2^ = 68.3% in the analysis of G2 and G3 chondrosarcoma) could be explained by the influence of the injected radiotracer dose, the time between injection and the initiation of detection, patient weight, body surface area, predictive cutoff value, detection equipment, and the individual characteristic of enrolled patients across included studies. Considering the differences of prognosis between benign cartilaginous lesions and G1 chondrosarcoma, as well as between G2 and G3 chondrosarcoma [3], SUVmax alone is not sufficient enough for grading chondrosarcoma and no matter what SUV-level of the cartilaginous lesions reveal, biopsy should not be resigned.

However, SUVmax did present crucial information for the management of chondrosarcoma. First, ^18^F-FDG PET can be performed in case that a high-grade chondrosarcoma is suspected by the initial manifestation or imaging (CT or MRI) in order to get a better tumor grading. Second, ^18^F-FDG PET scanning identifies tumor aggressiveness and extent visually and quantitatively, and therefore can direct the biopsy sampling by targeting the region of greatest SUVmax [29, 30]. This reduces the risk of false-negative results and re-biopsy as well as related complications. Third, SUVmax serves as a reference for image-guided percutaneous biopsy. For a cartilaginous lesion manifesting minimal likelihood of malignancy but high SUVmax, caution should be taken regarding the potential of elevating histologic grade based on pathological reports after operation. Last but not least, alteration of ^18^F-FDG avidity is an indication of relapse or sarcomatous transformation of cartilaginous neoplasms, and could be utilized in postoperative surveillance.

The relatively high price of ^18^F-FDG PET and PET/CT restricts its wide application in the clinical setting [12]. Normally, benign cartilaginous lesions and chondrosarcoma have certain different X-ray and clinical traits, which may provide information for the development of further diagnostic and therapeutic regimen (e.g., the necessity of biopsy) [2]. Therefore, ^18^F-FDG PET/CT should be performed only in cases with a high suspicion for malignant cartilaginous tumors (at the initial diagnosis, or when the possibility of malignant transformation of benign cartilaginous lesions or relapse of chondrosarcoma after treatment exists) prior to each biopsy, as a supplementary tool for assisting diagnosis and grading, guiding the fine needle aspiration and confirming the pathological result. MRI with contrast agent has ranked as a powerful method for the locoregional staging of malignant chondrosarcomas thanks to its spatial and contrast resolution, but it could not present metabolic data of the targeted lesions or be utilized for the whole-body assessment [31]. The current study has several limitations. The first is the absence of a standardized reference test for the diagnosis of chondrosarcoma. Either follow-up without an anatomopathological examination or biopsies may result in false negatives. The duration of follow-up differed from study to study, exceeding 12 months in most of the included ones but in Shin et al.’s investigation [22], the minimum follow-up was only 6 months. Another major limitation is that during the merging of the diagnostic data, subgroup analyses on the basis of important indicators such as types of chondrosarcoma, cutoff value, and study design were not conducted. Only 12 studies were pooled in the quantitative analysis and most (75%) [14–16, 20–22, 24–26] of them were retrospective. Third, sample sizes of the included studies were quite small and hence this meta-analysis might be subject to variability and inadequacy in data collection. Lastly, yet importantly, evidence of heterogeneity in data concerning ^18^F-FDG PET existed throughout included studies, and therefore we emphasized the pooled DOR, which was a global measure encompassing value of sensitivity, specificity, PLR, and NLR, as the main outcome of interest to compare the diagnostic performance of ^18^F-FDG PET and PET/CT.

## Conclusion

In a word, ^18^F-FDG PET/CT revealed excellent accuracy (pooled DOR = 112.999) in the diagnosis of chondrosarcoma and might assist in therapeutic decision-making. Although SUVmax alone showed restricted ability to differentiate benign cartilaginous lesions and G1 chondrosarcoma (pooled WMD = −0.89 95% CI, −1.67 to −0.10), as well as between G2 and G3 chondrosarcoma (pooled WMD = −2.37 95% CI, −5.79 to 1.05), it can identify intermediate/high-grade chondrosarcoma from low-grade ones (pooled WMD = −1.94 95% CI, −2.76 to −1.12). Cartilaginous lesions with high ^18^F-FDG-avidity should be highly monitored over the duration of treatment. More large-scale studies are still required to further warrant current findings.

## Data Availability

All data analyzed during this study are included in this published article.

## Abbreviations

CT: Computed tomography
MRI: Magnetic resonance imaging
18F-FDG: 18F-fluorodeoxyglucose
PET: Positron emission tomography
SUVmax: Maximum standardized uptake value
TP: True-positive
FP: False-positive
FN: False-negative
TN: True-negative
PLR: Positive likelihood ratio
NLR: Negative likelihood ratio
DOR: Diagnostic odds ratio
sROC: Summarized receiver operating characteristic
AUC: Area under curve
WMD: Weighted mean difference
CI: Confidence interval
